# Severity of omicron variant of concern and effectiveness of vaccine boosters against symptomatic disease in Scotland (EAVE II): a national cohort study with nested test-negative design

**DOI:** 10.1016/S1473-3099(22)00141-4

**Published:** 2022-07

**Authors:** Aziz Sheikh, Steven Kerr, Mark Woolhouse, Jim McMenamin, Chris Robertson, EAVE II Collaborators, Colin Richard Simpson, Tristan Millington, Ting Shi, Utkarsh Agrawal, Safraj Shahul Hameed, Elliott Hall, Igor Rudan, Syed Ahmar Shah, Lewis Ritchie, Sarah Stock, Colin McCowan

**Affiliations:** a Usher Institute, University of Edinburgh, Edinburgh, UK; b Public Health Scotland, Glasgow, UK; c Department of Mathematics and Statistics, University of Strathclyde, Glasgow, UK

## Abstract

**Background:**

Since its emergence in November, 2021, in southern Africa, the SARS-CoV-2 omicron variant of concern (VOC) has rapidly spread across the world. We aimed to investigate the severity of omicron and the extent to which booster vaccines are effective in preventing symptomatic infection.

**Methods:**

In this study, using the Scotland-wide Early Pandemic Evaluation and Enhanced Surveillance of COVID-19 (EAVE II) platform, we did a cohort analysis with a nested test-negative design incident case-control study covering the period Nov 1–Dec 19, 2021, to provide initial estimates of omicron severity and the effectiveness of vaccine boosters against symptomatic disease relative to 25 weeks or more after the second vaccine dose. Primary care data derived from 940 general practices across Scotland were linked to laboratory data and hospital admission data. We compared outcomes between infection with the delta VOC (defined as S-gene positive) and the omicron VOC (defined as S-gene negative). We assessed effectiveness against symptomatic SARS-CoV-2 infection, with infection confirmed through a positive RT-PCR.

**Findings:**

By Dec 19, 2021, there were 23 840 S-gene-negative cases in Scotland, which were predominantly among those aged 20–39 years (11 732 [49·2%]). The proportion of S-gene-negative cases that were possible reinfections was more than ten times that of S-gene-positive cases (7·6% *vs* 0·7%; p<0·0001). There were 15 hospital admissions in S-gene-negative individuals, giving an adjusted observed-to-expected admissions ratio of 0·32 (95% CI 0·19–0·52). The booster vaccine dose was associated with a 57% (54–60) reduction in the risk of symptomatic S-gene-negative infection relative to individuals who tested positive 25 weeks or more after the second vaccine dose.

**Interpretation:**

These early national data suggest that omicron is associated with a two-thirds reduction in the risk of COVID-19 hospitalisation compared with delta. Although offering the greatest protection against delta, the booster dose of vaccination offers substantial additional protection against the risk of symptomatic COVID-19 for omicron compared with 25 weeks or more after the second vaccine dose.

**Funding:**

Health Data Research UK, National Core Studies, Public Health Scotland, Scottish Government, UK Research and Innovation, and University of Edinburgh.

## Introduction

The omicron (B.1.1.529) SARS-CoV-2 variant of concern (VOC) was first detected in South Africa from a sample taken on Nov 9, 2021.^1^ This was reported to WHO on Nov 24, 2021, after which the WHO Technical Advisory Group on SARS-CoV-2 Virus Evolution was reconvened on Nov 26, 2021, leading to B.1.1.529 being denoted as a VOC.^2^ Omicron has subsequently spread globally.^3^

Omicron is characterised by several mutations of the spike protein.^4^ The small body of evidence available suggests that these mutations result in increased transmissibility when compared with the wild type and previous VOCs, and reduced potency of neutralising antibodies.^5^ Some studies have suggested tropism of omicron towards upper respiratory epithelial cells.^6^, ^7^ Early data from the ZOE COVID application suggest that the most common symptoms of omicron infection are mild and are those that are typically seen in upper respiratory tract infections.^8^ Several preliminary studies have found a reduced risk of hospitalisation for omicron relative to the delta variant (B.1.617.2) of SARS-CoV-2.^9^, ^10^, ^11^ However, further evidence is required on the severity of omicron infection, the extent to which previous infection with SARS-CoV-2 is protective, and the effectiveness of available COVID-19 vaccines in preventing disease.

In this study, we report our first estimates of the risk of hospital admission associated with omicron and the effectiveness of booster vaccinations compared with 25 weeks or more after second dose of vaccination in preventing symptomatic disease.

**Research in context**

**Evidence before this study**

We searched medRxiv, PubMed, and SSRN on Jan 31, 2022, using the terms “omicron”, “B.1.1.529”, and “vaccine effectiveness”, with no language restrictions. A preprint reported that two doses of BNT162b2 and ChAdOx1 were significantly less effective against the SARS-CoV-2 omicron variant (B.1.1.529) than against the delta variant (B.1.617.2). An analysis from the UK Health Security Agency found that omicron infection was significantly less likely to result in hospitalisation than delta infection and found high levels of booster dose effectiveness against omicron infection and hospitalisation. Studies using sera from vaccinated individuals have also found that the antibodies produced are less effective at neutralising omicron than delta. A paper from South Africa has suggested that omicron causes less severe disease than delta, which has at least in part been attributed to higher population immunity.

**Added value of this study**

This national investigation showed that the SARS-CoV-2 omicron variant was less likely to result in COVID-19 hospitalisation than was the delta variant. The study found that the rate of possible reinfection for omicron was ten times that of delta. We also found that third booster vaccine doses offered considerable additional protection against symptomatic disease when compared with 25 weeks or more after the second vaccine dose, with these benefits seen for all vaccines administered in the UK.

**Implications for all the available evidence**

These findings provide evidence for the acceleration and extension of the vaccine booster programme. Although these are early observations of reduced severity of omicron relative to delta in risk of hospitalisation, the findings are encouraging. The combination of an increased risk of transmission and immune evasion of omicron might mean that any advantage in reduced hospitalisation could potentially be exceeded by increased rates of infection in the community. Incorporation of our data on the risk of hospitalisation within modelling output could inform decisions by policy makers regarding the speed, range, nature, and duration of societal measures that otherwise would be needed to control the risk of spread of infection and minimise the risk of overwhelming health system capacity.

## Methods

### Study design and population

Our methods have been described in detail in a number of previous publications.^12^, ^13^, ^14^, ^15^ We used a Scotland-wide prospective cohort, which comprises linked datasets on 5·4 million people in Scotland (around 99% coverage), to construct a nested test-negative design study among individuals with incident symptomatic infections.

Primary care data derived from 940 general practices across Scotland were linked to laboratory data and hospital admission data available from the Rapid Preliminary Inpatient Data (RAPID) dataset.^16^ Vaccination data were available from general practices and the Turas Vaccination Management Tool.^17^ Laboratory data from the Electronic Communication of Surveillance in Scotland system included all RT-PCR results from UK National Health Service (NHS) laboratories (pillar 1) and the Lighthouse laboratory (pillar 2).^18^ Data were deterministically linked using the unique Community Health Index number.

Information on S-gene status was available from individuals tested in the community by the Lighthouse laboratory. Most hospital admissions arose from those tested in the NHS laboratories and S-gene information was not routinely available from those laboratories. There is a national sequencing surveillance system, and a representative sample of positive cases are sequenced, with around 2000 samples sequenced each week. There is a delay of about 2 weeks for the sequencing results.

The main analysis in this report is based upon all patients who tested positive from the community in Scotland between Nov 1 and Dec 19, 2021, with follow-up from the date of testing positive to the date of hospital admission. The hospitalisation analysis used the last date of admission to hospital (ie, Dec 21, 2021); follow-up was censored at 15 days after diagnosis. In the test-negative design, the first positive test result after the beginning of the study was used for individuals with at least one positive test. For individuals with multiple negative tests, one test was selected at random. Only individuals reporting symptoms at the time of test were included in this study and the date of symptom onset was used. A small number of individuals reported symptoms with no date of onset and this was imputed as 5 days before the test.

Approvals for the study were obtained from the National Research Ethics Service Committee, Southeast Scotland 02 (reference number 12/SS/0201) and Public Benefit and Privacy Panel for Health and Social Care (reference number 1920-0279).

We followed the reporting of studies conducted using observational routinely collected data checklist to guide transparent reporting of this study. Our analysis code is publicly available.

### Exposure definitions

We studied second doses of BNT162b2 (Pfizer-BioNTech),^19^ ChAdOx1 (Oxford-AstraZeneca),^20^ and mRNA-1273 (Moderna)^12^ vaccines, and third or booster doses of BNT162b2 and mRNA-1273. Vaccination status was defined on the date of the positive RT-PCR (symptom date for test-negative design) test and coded using the following categories: unvaccinated; 1–27 days after first dose; 28 days or more after first dose; 0–13 days after second dose; 14–41 days after second dose; 42–69 days after second dose; and 10 weeks or more (≥70 days) after second dose. For those with a third or booster dose, the categories were 0 or 1 week after booster dose or 2 or more weeks after booster dose.

Vaccinated groups were stratified by time intervals since second and third doses of vaccine and whether infection was caused by delta (S-gene positive) or omicron (S-gene negative). The S-gene variable took one of five values, as follows: S-positive (delta), weak S-positive (usually also delta), S-negative (omicron), other, and unknown. Unknown corresponded to individuals who were tested in NHS laboratories (where S-gene status was unavailable) or who were tested in the Lighthouse laboratory, but the sample did not yield any cycle threshold values. Other corresponded to cycle threshold values that could not otherwise be classified.

### Definition of outcomes

We assessed effectiveness against symptomatic SARS-CoV-2 infection, with infection confirmed through a positive RT-PCR.

COVID-19 hospitalisation was defined as an emergency admission to hospital in individuals with a positive RT-PCR test 14 days or less before admission or who tested positive within 2 days of admission. Patients who were already in hospital and then tested positive more than 2 days after admission were excluded from the analysis. Hospital admission data came from the RAPID database. Details of admission and discharge codes are available from Scottish Morbidity Record 01—hospital admissions and discharges, but this has a 2-month delay for validation. Public Health Scotland has done an audit in three health boards for the period Dec 20, 2021, to Jan 11, 2022, which found that 63% of hospital admissions were due to COVID-19, as opposed to with COVID-19, where SARS-CoV-2 infection was not the primary reason for admission to hospital.^22^

### Patient characteristics and confounders

We considered the following potential confounders: socio-economic status measured by quintiles of the Scottish Index of Multiple Deprivation (1 refers to most deprived and 5 refers to least deprived), residential settlement measured by the urban rural 6-fold classification (1 refers to large urban areas and 6 refers to small remote rural areas), the number and types of comorbidities commonly associated with COVID-19,^23^ and whether the individual had a previous positive SARS-CoV-2 test. Age and sex were recorded at the date of test and date of vaccination.

### Statistical analysis

The expected numbers of COVID-19 hospitalisations were calculated by fitting a Cox proportional hazards model to the S-gene-positive cases only in the study period using predictors of age group, sex, deprivation status, previous positive test history, number of co-morbid QCOVID clinical risk groups (0, 1, 2, 3, 4, or ≥5),^23^ and vaccine status, including vaccine type, dose, and duration, as well as a calendar period effect in weeks. QCOVID is a tool for predicting the risk of COVID-19 hospitalisation and death that has been used to inform UK policies on vaccine prioritisation. The expected number of cases was derived from the predictions of expected survival from the model. Hence, the expected number of hospitalisations in the S-gene-positive group matched the observed number of cases. CIs were derived using Byar's method.^24^

Analysis of the risk of symptomatic disease was by generalised additive logistic regression, including spline terms for age and the temporal trend during the study period. All models included vaccine status. Further adjustment was made for health board, sex, deprivation, previous positive test history, number of QCOVID clinical risk groups, and whether the individual was recorded as being immunosuppressed or in a shielding category. This analysis was done separately for those aged 16–49 years and those aged 50 years and older to assess any differential reduction in risk and because most unvaccinated people were in the younger age group.

In the test-negative design, the reduction in the odds ratio (OR) of testing S-gene-negative or S-gene-positive after receipt of a booster or third dose of any vaccine was measured relative to individuals who had received two vaccine doses 25 weeks or more before symptom onset. There were three reasons for this methodology. First, there were very few unvaccinated individuals, particularly among older adults, and so the precision of estimates of vaccine effectiveness relative to unvaccinated individuals would be low. Second, associated with the low numbers in the unvaccinated group is potential bias in the unvaccinated group in a population in which most people were vaccinated and the two doses at 25 weeks or more group represented those who were initially targeted for the booster dose. Third, studies have shown significant vaccine waning by 25 weeks or more after the second dose, although waning might not be homogenous across the population.^25^

The previous positive test variable included categories for whether an individual had an RT-PCR-confirmed test before their test in the study period. The categories were as follows: never positive, positive 1–28 days before symptom onset in the test-negative design, positive 29–90 days before, and positive more than 90 days before. Possible reinfection was defined as a positive RT-PCR test more than 90 days after an initial positive RT-PCR.

Six sensitivity analyses were done for the calculation of the expected number of hospitalisations. First, only individuals with at least 7 days follow-up after testing positive were included in the analysis, as most admissions from the community to hospital will have occurred by that time among those with S-gene-positive infection. Second, most individuals with S-gene-negative infection were aged 20–59 years, so we did a sub-analysis in this group only. Third, a small percentage of those who tested positive did not link into the Early Pandemic Evaluation and Enhanced Surveillance of COVID-19 (EAVE II) platform, and although we knew their age, sex, and vaccine and testing status, their QCOVID risk groups and deprivation status were unknown; therefore for these individuals only, the number of risk groups was imputed as the modal value 0 and deprivation status was imputed as level 3, the middle quintile. The fourth sensitivity analysis used a less complex prediction model, including age group, sex, number of co-morbid QCOVID clinical risk groups, and vaccine status, including dose and duration, and calendar period. The fifth sensitivity analysis used stratification by age group rather than adjustment by age. The sixth sensitivity analysis included stratification by both age group and number of risk groups.

Analyses were done with R (version 3.6.1). Analyses were carried out by CR and independently checked by SK.

### Role of the funding source

The funders had no role in study design, data collection, data analysis, data interpretation, or writing of the report.

## Results

Between Nov 1 and Dec 19, 2021, there were 162 946 positive RT-PCR tests. The characteristics of those testing positive by S-gene status is summarised in the appendix (p 1). 152 496 (93·6%) of 162 946 test results were available to us. The rate of S-gene-positive infection was greater among the unvaccinated population, who were mainly children (aged under 16 years), whereas 11 732 (49·2%) of 23 840 with S-gene-negative infection were aged 20–39 years (appendix pp 1, 7–8).

856 (95·5%) of 896 patients hospitalised within 14 days of a community test were S-gene positive and 15 (1·7%) were S-gene negative; 455 (50·8%) were female and 441 (49·2%) were male; and 54 (6·0%) were younger than 20 years, 205 (22·9%) were aged 20–39 years, 396 (44·4%) were aged 40–59 years, and 241 (26·9%) were aged 60 years and older. There were 45 COVID-19-related deaths, all in the S-gene-positive group.

The rate of possible reinfection for S-gene-negative infection was around ten times that of S-gene-positive infection (1800 [7*·*6%] of 23 840 cases *vs* 948 [0*·*7%] of 126 511 cases; p<0·0001; appendix p 1), but there was evidence of protection from omicron infection within the test-negative design where the OR of testing positive for S-gene-negative infection among those previously positive more than 90 days before the symptomatic test was 0*·*57 (95% CI 0*·*53–0*·*61); for an S-gene-positive infection the corresponding OR was 0*·*07 (0*·*07–0*·*08).

Among the community-tested individuals, 686 (99·8%) of 687 omicron cases were S-gene-negative and, of community-tested individuals whose sample was S-gene-negative and sequenced, 686 (97·9%) of 701 cases were omicron. 22 616 (99·9%) of 22 637 community-tested delta cases were S-gene positive and 22 616 (99·9%) of 22 640 community S-gene-positive cases were delta (appendix p 3).

Trends in hospital admission among those testing positive for SARS-CoV-2 from Nov 1, 2021, showed that most admissions were associated with S-gene-positive infection and very few were associated with S-gene-negative infection (appendix p 9). Hospitalisation rates by age group are shown in the appendix (p 10), showing lower admission rates in adults aged 20–59 years who had S-gene-negative infection compared with S-gene-positive infection.

There was a lower than expected number of hospital admissions for COVID-19 in individuals with S-gene-negative infection (table 1). The adjusted observed-to-expected ratio was 0·32 (95% CI 0·19–0·52). Using the entire cohort (ie, including the relatively few cases that could not be linked) yielded a comparable observed-to-expected ratio of 0·36 (0·22–0·56; table 1). Stratifying by age, and by age and number of risk groups, had little effect on the results. Cumulative incidence curves are presented in the figure and the hazard ratios (HRs) from the Cox model are presented in the appendix (p 4).

**Table 1:** Observed versus expected analysis for risk of hospital admission by S-gene status

	**N**	**Person-years**	**Hospital admissions**	**Expected admissions**	**Observed-to-expected ratio**	**95% CI**
**All cases linking into the EAVE dataset**						
S-gene-positive	119 100	4375·1	856 (0·72%)	856·9	1·00	0·93–1·07
S-gene-negative	22 205	413·4	15 (0·07%)	46·6	0·32	0·19–0·52
Weak S-gene-positive	2199	57·3	7 (0·32%)	6·9	1·02	0·45–2·00
Other	990	33·8	..	..	0·79	0·26–1·88
Unknown	1647	58·2	14 (0·85%)	14·8	0·94	0·54–1·54
**All cases with imputation**						
S-gene-positive	126 464	4643·5	967 (0·76%)	903·7	1·07	1·00–1·14
S-gene-negative	23 830	443·1	18 (0·08%)	50·1	0·36	0·22–0·56
Weak S-gene-positive	2384	62·1	9 (0·38%)	7·5	1·20	0·59–2·19
Other	1080	36·5	..	..	0·71	0·24–1·69
Unknown	1813	63·3	17 (0·94%)	16·1	1·05	0·64–1·65
**All linked cases followed up for at least 7 days**						
S-gene-positive	102 765	4096·2	824 (0·80%)	824·9	1·00	0·93–1·07
S-gene-negative	4111	140·2	7 (0·17%)	21·2	0·33	0·15–0·65
Weak S-gene-positive	995	37·5	7 (0·70%)	5·3	1·32	0·59–2·59
Other	748	29·5	..	..	0·64	0·18–1·70
Unknown	1336	52·8	10 (0·75)	14·1	0·71	0·36–1·25
**All linked cases aged 20–59 years**						
S-gene-positive	68 035	2489·4	575 (0·85%)	575·6	1·00	0·92–1·08
S-gene-negative	17 302	322·9	15 (0·09%)	34·4	0·44	0·25–0·70
Weak S-gene-positive	1373	34·7	6 (0·44%)	5·1	1·18	0·49–2·44
Other	567	19·1	..	..	0·58	0·11–1·85
Unknown	1057	36·4	5 (0·47%)	8·6	0·58	0·22–1·28
**All linked cases using simpler prediction model**						
S-gene-positive	119 100	4375·1	856 (0·72%)	856·9	1·00	0·93–1·07
S-gene-negative	22 205	413·4	15 (0·07%)	52·4	0·29	0·17–0·46
Weak S-gene-positive	2199	57·3	7 (0·32%)	8·0	0·88	0·39–1·72
Other	990	33·8	..	..	0·75	0·25–1·79
Unknown	1647	58·2	14 (0·85%)	16·3	0·86	0·49–1·40
**All linked cases using stratification by age group**						
S-gene-positive	119 100	4375·1	856 (0·72%)	856·9	1·00	0·93–1·07
S-gene-negative	22 205	413·4	15 (0·07%)	48·1	0·31	0·18–0·50
Weak S-gene-positive	2199	57·3	7 (0·32%)	7·2	0·98	0·44–1·92
Other	990	33·8	..	5·1	0·78	0·26–1·85
Unknown	1647	58·2	14 (0·85%)	15·1	0·93	0·53–1·52
**All linked cases stratified by age group and number of risk groups**						
S-gene-positive	119 100	4375·1	856 (0·72%)	856·9	1·00	0·93–1·07
S-gene-negative	22 205	413·4	15 (0·07%)	48·7	0·31	0·18–0·50
Weak S-gene-positive	2199	57·3	7 (0·32%)	7·3	0·96	0·43–1·88
Other	990	33·8	..	5·2	0·77	0·26–1·83
Unknown	1647	58·2	14 (0·85%)	15·2	0·92	0·53–1·50

N is the number of individuals who tested positive. Person-years is the total follow-up time from testing positive. Hospital admissions are the number of people admitted to hospital for at least 1 day within 14 days of a positive test. 95% CI shows the lower and upper confidence intervals for the observed-to-expected ratio based upon a Poisson distribution for the admissions. As the model is fitted to the S-gene-positive data the observed and expected will match exactly. The table gives the expected number of hospital admissions for the other S-gene categories assuming that the observed pattern among the S-gene-positive cases applies. The model included adjustments for age group, sex, deprivation status, previous positive SARS-CoV-2 test, number of comorbid QCOVID clinical risk groups, and vaccine status, including vaccine type, dose, and duration, as well as a calendar period effect in weeks. Cells with missing data indicate small numbers of admissions that have been suppressed (we do not have permission to disclose actual numbers if the cell count is <5), alongside the expected values.

**Figure:**
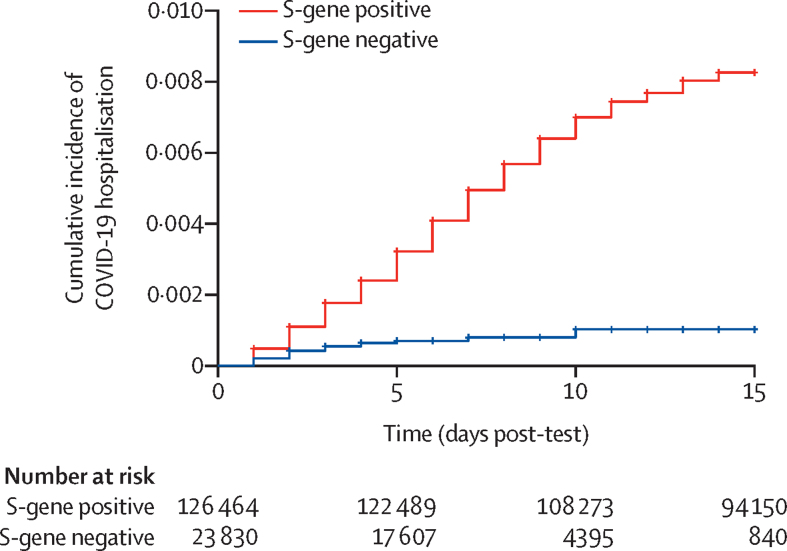
Cumulative incidence of COVID-19 hospitalisation by S-gene status

The sensitivity analyses for the expected numbers of hospitalisations showed that using only those who had been followed up for 7 days or more had a similar observed-to-expected ratio to the primary analysis for S-gene-negative infection of 0·33 (95% CI 0·15–0·65; table 1). When considering those aged 20–59 years, the ratio was slightly higher at 0·44 (0·25–0·70), but with overlapping, though imprecise, 95% CIs. Additional sensitivity analyses used a reduced statistical model that did not include vaccine type, deprivation, and previous tests, giving an observed-to-expected ratio of 0·29 (0·17–0·46), whereas stratifying the time to hospital admission curve by age group yielded a ratio of 0·31 (0·18–0·50). Stratifying by age group and number of risk groups gave a ratio of 0·31 (0·18–0·50).

Relative to 25 weeks or more after second vaccination, a third or booster vaccine dose was associated with a 56% (95% CI 51–60) reduction in the odds of developing symptomatic disease with S-gene-negative infection 2 or more weeks after booster vaccination among those aged 16–49 years (table 2). For individuals aged 50 years and older, the corresponding reduction was 57% (52–62). Across all age groups, the reduction in developing symptomatic disease was 57% (54–60). These reductions in the odds of infection were lower than for symptomatic S-gene-positive infection, where booster vaccination was associated with an 83% (95% CI 81–84) reduction in developing symptomatic disease in those aged 16–49 years and an 88% reduction (86–89) in those aged 50 years and older.

**Table 2:** Effectiveness of booster vaccinations for symptomatic positive S-gene-negative test compared with individuals who had two doses of vaccine ≥25 weeks before testing positive

		**S-gene-negative infections**			**S-gene-positive infections**		
		Tested, n	Positive, n	Relative vaccine effectiveness, % (95% CI)	Tested, n	Positive, n	Relative vaccine effectiveness, % (95% CI)
**16–49 years**							
Unvaccinated		10 302	1003	22% (14 to 29)	14 583	5284	−98% (−109 to −87)
First dose							
	0–27 days	550	36	47% (24 to 63)	676	162	−24% (−50 to −3)
	≥28 days	6570	581	30% (21 to 38)	8339	2350	−39% (−49 to −30)
Second dose							
	0–13 days	732	46	58% (42 to 70)	805	119	31% (16 to 44)
	14–69 days	4248	256	53% (46 to 60)	4258	266	73% (69 to 76)
	70–104 days	12 581	814	33% (26 to 40)	13 559	1792	50% (46 to 53)
	105–139 days	29 209	3503	15% (9 to 21)	31 963	6257	32% (29 to 36)
	140–174 days	14 986	1824	3% (−5 to 11)	17 991	4829	9% (4 to 13)
	≥175 days	13 183	1435	Reference	15 462	3714	Reference
Third dose							
	0–6 days	3773	515	26% (16 to 34)	4003	745	33% (27 to 39)
	7–13 days	2185	143	62% (54to 68)	2155	113	84% (80 to 87)
	≥14 days	12 887	783	56% (51 to 60)	12 798	694	83% (81 to 84)
**≥50 years**							
Unvaccinated		716	48	33% (7 to 52)	1158	490	−45% (−65 to −28)
First dose							
	0–27 days	27	4	0 (−230 to 70)	36	13	−16% (−134 to 42)
	≥28 days	256	13	48% (7 to 72)	343	100	10% (−15 to 30)
Second dose							
	0–13 days	23	1	62% (−207 to 95)	23	1	90% (27 to 99)
	14–69 days	120	9	5% (−98 to 54)	131	20	62% (38 to 77)
	70–104 days	128	12	8% (−76 to 52)	149	33	40% (10 to 60)
	105–139 days	463	17	35% (−10 to 62)	634	188	20% (4 to 33)
	140–174 days	5513	265	4% (−13 to 19)	8205	2957	4% (−3 to 10)
	≥175 days	8007	799	Reference	10 856	3648	Reference
Third dose							
	0–6 days	3522	420	0 (−15 to 13)	4352	1250	20% (13 to 26)
	7–13 days	3006	180	54% (46 to 62)	3146	320	77% (74 to 80)
	≥14 days	17 572	1045	57% (52 to 62)	17 504	977	88% (86 to 89)

Effectiveness of vaccine boosters is measured as 1 – odds ratio. Vaccination status gives the number of weeks since most recent dose. Tested indicates the number of symptomatic individuals who were tested in the analysis and positive indicates the number who tested positive. The number who tested negative for SARS-CoV-2 infection is the difference between the tested and positive and this is the same in both the S-gene-negative and S-gene-positive analysis.

Within these analyses, adjustment was made for the effect of a previous positive test. For symptomatic individuals, the OR of an S-gene-positive infection having been positive more than 90 days before symptom onset was 0·08 (95% CI 0·07–0·09); for testing positive 28–90 days before symptom onset the OR was 0·06 (0·05–0·08). The corresponding ORs for S-gene-negative infection were 0·57 (0·53–0·61) and 0·25 (0·20–0·32), respectively.

## Discussion

Omicron has spread rapidly across a highly vaccinated population in Scotland, replacing delta as the dominant VOC in less than a month. Although preliminary, our data suggest that omicron is substantially less likely to result in COVID-19 hospitalisation than is delta. We also showed that a third or booster dose of vaccine was associated with considerable additional protection against symptomatic infection within 2 weeks of this additional dose compared with two doses of vaccine received 25 weeks ago or more. This protection is greatest for delta, but still substantial for omicron.

A key strength of this study is our use of national linked datasets, thereby reducing the risk of selection, recall, and misclassification biases. Our study also has limitations. First, we used the surrogate of S-gene status as a marker of delta or omicron infection. Sequencing data were only available for a subset of the population and there was a lag in obtaining these data. However, our data show that these proxies are likely to be reliable markers, with more than 99% of S-gene-positive cases being sequenced as delta and 98% of S-gene-negative cases being sequenced as omicron (appendix p 3). Another limitation is that S-gene status could only be determined in those tested in the community, since the Taq-Path assay was only available in the Lighthouse laboratory that processed all community tests, so we are unable to comment on those testing positive in hospital settings. Therefore, our results were only representative of community-based cases. However, such individuals are likely to be healthier than those admitted to hospital after a positive test in NHS laboratories. There were too few serious COVID-19 outcomes in those who were S-gene negative to enable analysis of the effectiveness of booster vaccination against COVID-19 deaths. Finally, because of the low number of hospital admissions, there was considerable uncertainty in the estimation of the observed-to-expected ratios.

The modelling analysis relied on several assumptions. First, that the pattern of time to hospital admission from the community after a positive test is the same for S-gene-negative infections and S-gene-positive infections. It will take time to assess if this is indeed the case. Second, there was little circulation of S-gene-negative infections among older people in this early part of the epidemic. If hospitalisation rates with omicron among older people are higher than with delta, this will lead to overly optimistic conclusions from this early report. Another issue is that we had little data on time since booster dose of the vaccine, and if there is waning after the booster this will effect hospitalisations, particularly in older people who received their booster doses in autumn 2021. Finally, most hospital admissions come from individuals tested in NHS laboratories in Scotland, and this analysis does not cover these admissions.

Several reports have indicated increased transmission associated with omicron compared with delta, which has resulted in considerable concern among governments, public health officials, and the general public over the risk that health system capacity will be breached. These concerns have been compounded by data showing a reduced effect associated with two doses of vaccine and reduced neutralising antibodies against omicron compared with delta, suggesting increased potential for vaccine escape.^26^, ^27^, ^28^ The available modelling, which has assumed comparable severity for omicron and delta, suggests that in most scenarios there will be a very sharp increase in the number of hospital admissions and deaths as omicron begins to replace delta. A key gap in the evidence base has been the absence of data on the severity of disease associated with omicron, which has led to several governments beginning to reimpose social distancing restrictions. Early data from South Africa suggest that omicron is associated with a reduced risk of severe disease.^11^ Our findings are consistent with this, and subsequent UK studies.^9^, ^10^ Our data should reduce the uncertainty in this key parameter used to model the impact of the growth of omicron. The reduced risk of severe disease had implications for isolation rules, which were in place in the UK in winter 2021–22. These isolation rules can contribute to the inadvertent closing down of society, as ever-increasing numbers of people get infected and need to isolate, threatening the viability of essential services such as the NHS and public transport. Our findings of decreased severity suggest that omicron might signal the UK entering an endemic phase of COVID-19. This theory is supported by a report from Public Health Scotland covering the 5-week period to Dec 20, 2021, which found that 89·7% of people attending community health-care services had COVID-19 antibodies.^29^

A further piece of information identified in this study is the proportion of cases identified as possible reinfections, which can be factored into modelling outputs. The combination of increased risk of transmission and immune evasion of omicron might mean that any advantage in reduced hospitalisation could potentially be exceeded by increased rates of infection in the community. However, our analysis focused on COVID-19 cases in the period from Nov 1 to Dec 19, 2021. Delta cases in this period might have been less severe than in earlier periods. Therefore, the difference in the risk of hospitalisation that we estimated might be smaller than would have been obtained by using earlier delta cases as the comparator.

In our study population, S-gene-positive infections were more concentrated in younger and unvaccinated individuals than were S-gene-negative infections. This finding might be partly explained by the delta variant having been in circulation for longer than the omicron variant. In our test-negative design study, we found evidence of substantial protection against symptomatic infection with omicron. The level of protection waned over time, and we found that after around 15 weeks since a second vaccine there was a greater risk of symptomatic infection compared with unvaccinated individuals. This finding is unusual in vaccine effect studies and the most likely explanation is residual confounding, which has not been fully adjusted for with different exposure patterns in the vaccinated and unvaccinated groups. An alternative explanation could be that in the early stages of omicron's arrival into Scotland, the variant initially spread around those aged 20–39 years, many of whom were double vaccinated around 3–4 months previously. Believing that they were still protected, individuals might have increased social contact. Of relevance is that omicron infection had largely not yet reached younger age groups, most of whom were unvaccinated and who might therefore have been taking more precautions. A similar effect has also been reported in other studies.^30^

In conclusion, although preliminary, these national data provide some reassurance that omicron is substantially less likely to result in severe outcomes than delta and that third or booster vaccine doses are associated with considerable added protection against symptomatic disease when compared with second vaccine doses. We will continue to analyse the Scottish data, which should lead to greater precision in our estimates over the coming weeks and months. The policy implications of these findings are potentially substantial, but there is a need for confirmatory findings from research groups in other countries.

## Data sharing

A data dictionary covering the datasets used in this study can be found at https://github.com/EAVE-II/EAVE-II-data-dictionary. All code used in this study is publicly available at https://github.com/EAVE-II/B.1.1.529-variant. The data used to undertake this analysis are not publicly available because they are based on deidentified national clinical records. These data are available, subject to approval by the NHS Scotland Public Benefit and Privacy Panel, by application through the Scotland National Safe Haven (https://www.informationgovernance.scot.nhs.uk/pbpphsc/).

## Declaration of interests

AS, MW, CR, and JM are members of the Scottish Government Chief Medical Officer's COVID-19 Advisory Group and AS is a member of its Standing Committee on Pandemics. AS and JM are also members of the New and Emerging Respiratory Virus Threats Advisory Group (NERVTAG). JM is the Chair of the multidisciplinary Scottish COVID-19 National Incident Management Team. AS is a member of AstraZeneca's Thrombotic Thrombocytopenic Taskforce. All AS’ roles are unremunerated. CR and MW are members of the Scientific Pandemic Influenza Group on Modelling. SK declares no competing interests.
