# Supplementary material for: Severity of omicron variant of concern and effectiveness of vaccine boosters against symptomatic disease in Scotland (EAVE II): a national cohort study with nested test-negative design
Source: Lancet Infect Dis. 2022 Jul;22(7):959–66. doi: 10.1016/S1473-3099(22)00141-4 (PMC9033213; doi:10.1016/S1473-3099(22)00141-4)

THE LANCET
Infectious Diseases

Supplementary appendix

This appendix formed part of the original submission and has been peer reviewed. We post it as supplied by the authors.

Supplement to: Sheikh A, Kerr S, Woolhouse M, et al. Severity of omicron variant of concern and effectiveness of vaccine boosters against symptomatic disease in Scotland (EAVE II): a national cohort study with nested test-negative design. *Lancet Infect Dis* 2022; published online April 22. https://doi.org/10.1016/S1473-3099(22)00141-4.

Supplementary materials

Table S1: Characteristics of those testing positive by S gene status

Characteristic	Levels	S Positive	S Negative	Weak S Positive	Other	Unknown
Total		126,511	23,840	2,385	1,081	9,129
Sex	Female	65,128 (51.5%)	12,805 (53.7%)	1,355 (56.8%)	553 (51.2%)	4,659 (51.0%)
	Male	61,383 (48.5%)	11,035 (46.3%)	1,030 (43.2%)	528 (48.8%)	4,470 (49.0%)
Age group	0-11	29,329 (23.2%)	1,389 (5.8%)	500 (21.0%)	253 (23.4%)	1,238 (13.6%)
	12-19	14,665 (11.6%)	2,277 (9.6%)	253 (10.6%)	126 (11.7%)	549 (6.0%)
	20-39	32,628 (25.8%)	11,732 (49.2%)	879 (36.9%)	352 (32.6%)	2,037 (22.3%)
	40-59	39,528 (31.2%)	6,862 (28.8%)	609 (25.5%)	278 (25.7%)	2,535 (27.8%)
	60-74	9,101 (7.2%)	1,354 (5.7%)	126 (5.3%)	66 (6.1%)	1,401 (15.3%)
	75+	1,260 (1.0%)	226 (0.9%)	18 (0.8%)	6 (0.6%)	1,369 (15.0%)
Number of risk groups	0	86,753 (68.6%)	15,888 (66.6%)	1,634 (68.5%)	736 (68.1%)	4,553 (49.9%)
	1	25,341 (20.0%)	5,206 (21.8%)	457 (19.2%)	208 (19.2%)	1,911 (20.9%)
	2	5,410 (4.3%)	888 (3.7%)	91 (3.8%)	34 (3.1%)	863 (9.5%)
	3	1,206 (1.0%)	170 (0.7%)	12 (0.5%)	10 (0.9%)	456 (5.0%)
	4	352 (0.3%)	39 (0.2%)	5 (0.2%)	* (0.1%)	256 (2.8%)
	5+	151 (0.1%)	23 (0.1%)	* (0.0%)	* (0.1%)	216 (2.4%)
	Unknown	7,298 (5.8%)	1,626 (6.8%)	185 (7.8%)	91 (8.4%)	874 (9.6%)
Vaccine status	Unvaccinated	47,972 (37.9%)	3,548 (14.9%)	712 (29.9%)	408 (37.7%)	2,749 (30.1%)

First dose	0-27 days	965 (0.8%)	123 (0.5%)	18 (0.8%)	8 (0.7%)	54 (0.6%)
	28+ weeks	9,323 (7.4%)	1,460 (6.1%)	145 (6.1%)	89 (8.2%)	528 (5.8%)
Second dose	0-13 days	311 (0.2%)	88 (0.4%)	13 (0.5%)	5 (0.5%)	25 (0.3%)
	14-41 days	219 (0.2%)	127 (0.5%)	15 (0.6%)	7 (0.6%)	23 (0.3%)
	42-69 days	728 (0.6%)	258 (1.1%)	28 (1.2%)	6 (0.6%)	37 (0.4%)
	70+ days	56,099 (44.3%)	12,612 (52.9%)	943 (39.5%)	435 (40.2%)	3,816 (41.8%)
Third dose	0-13 days	6,351 (5.0%)	2,364 (9.9%)	188 (7.9%)	58 (5.4%)	664 (7.3%)
	14+days	4,543 (3.6%)	3,260 (13.7%)	323 (13.5%)	65 (6.0%)	1,233 (13.5%)
Previously tested positive	Never	125,064 (98.9%)	21,949 (92.1%)	2,123 (89.0%)	1,034 (95.7%)	8,285 (90.8%)
	1 to 28 days before	292 (0.2%)	0 (0.0%)	* (0.2%)	* (0.4%)	375 (4.1%)
	29 to 90 days before	207 (0.2%)	91 (0.4%)	39 (1.6%)	10 (0.9%)	249 (2.7%)
	> 90 days before	948 (0.7%)	1,800 (7.6%)	219 (9.2%)	33 (3.1%)	220 (2.4%)

The numbers in some cells are suppressed to avoid counts of less than 5, denoted *.

Number of risk groups is derived from the QCOVID algorithm categories. The individuals whose comorbid status is unknown are those who did not link into the EAVE II study. This can occur if a person recently moved into Scotland or was not registered with a GP practice in December 2020,

The majority of individuals whose S gene status is unknown tested positive in an NHS laboratory and S Gene status is not routinely available. They are included for completeness but are not used in any of the modelling analysis.

Table S2: Number of samples with S gene status by sequencing variant

S gene status	Delta	Omicron	Other	Not sequenced	Total
**S gene positive**	22,616	1	23	103,871	126,511
**S gene negative**	10	686	5	23,139	2,3840
**Weak S positive**		0	0	2,381	2,385
**Other**	11	0	0	1,070	1,081
**Unknown**	2,819	18	17	6,275	9,129
**Total**	25,460	705	45	136,736	162,946

The information in this table is based upon sequencing information available up to December 11, 2021. The majority of individuals whose S gene status is unknown tested positive in an NHS laboratory and S gene status is not routinely available. A greater proportion of NHS laboratory cases are sequenced compared to community cases. Some cells have small numbers of admissions and these have been suppressed (*) as well as the expected values.

Table S3: Numbers, percentages and hazard ratios for each characteristic included in the Cox regression model

Characteristic	Levels	S positive	Hospitalised	HR (95% CI)	S negative
Week	1	17,030 (14.3%)	174 (20.3%)	1	10 (0.0%)
	2	18,902 (15.9%)	173 (20.2%)	0.98 (0.79 – 1.21)	5 (0.0%)
	3	18,056 (15.2%)	141 (16.5%)	0.86 (068 – 1.07)	8 (0.0%)
	4	15,587 (13.1%)	112 (13.1%)	0.83 (0.65 – 1.05)	73 (0.3%)
	5	17,055 (14.3%)	141 (16.5%)	0.99 (0.79 – 1.24)	436 (2.0%)
	6	18,181 (15.3%)	91 (10.6%)	0.67 (0.52 – 0.87)	4,450 (20.0%)
	7	14,289 (12.0%)	24 (2.8%)	0.43 (0.28 – 0.67)	17,223 (77.6%)
Age	0-11	27,282 (22.9%)	36 (4.2%)	1	1,244 (5.6%)
	12-19	13,906 (11.7%)	15 (1.8%)	1.14 (0.62 – 2.11)	2,140 (9.6%)
	20-39	30,410 (25.5%)	193 (22.5%)	9.12 (6.32 – 13.18)	10,801 (48.6%)
	40-59	37,625 (31.6%)	382 (44.6%)	16.72 (11.53 – 24.25)	6,501 (29.3%)
	60-74	8,695 (7.3%)	181 (21.1%)	28.67 (19.22 – 42.75)	1,302 (5.9%)
	75+	1,182 (1.0%)	49 (5.7%)	38.56 (23.43 – 63.48)	217 (1.0%)
Sex	Female	61,556 (51.7%)	429 (50.1%)	1	11,997 (54.0%)
	Male	57,544 (48.3%)	427 (49.9%)	1.07 (0.93 – 1.22)	10,208 (46.0%)
Deprivation	1 - High	21,995 (18.5%)	228 (26.6%)	1	3,811 (17.2%)
	2	23,188 (19.5%)	194 (22.7%)	0.87 (0.72 – 1.05)	3,993 (18.0%)
	3	22,537 (18.9%)	166 (19.4%)	0.80 (0.65 – 0.98)	3,862 (17.4%)
	4	24,687 (20.7%)	141 (16.5%)	0.67 (0.54 – 0.83)	4,654 (21.0%)
	5-Low	25,892 (21.7%)	124 (14.5%)	0.62 (0.50 – 0.78)	5,688 (25.6%)
Number of co-morbid conditions	0	86,717 (72.8%)	364 (42.5%)	1	15,884 (71.5%)
	1	25,304 (21.2%)	272 (31.8%)	1.90 (1.62 – 2.23)	5,204 (23.4%)
	2	5,398 (4.5%)	130 (15.2%)	3.41 (2.77 – 4.20)	886 (4.0%)
	3	1,193	56	5.45	170

		(1.0%)	(6.5%)	(4.05 – 7.32)	(0.8%)
	4	346 (0.3%)	22 (2.6%)	6.17 (3.93 – 9.70)	39 (0.2%)
	5+	142 (0.1%)	12 (1.4%)	7.12 (3.91 – 12.98)	22 (0.1%)
Vaccine status and type for first 2 doses	uv	44,091 (37.0%)	264 (30.8%)	1	2,944 (13.3%)
	v1_4+_AZ	798 (0.7%)	10 (1.2%)	0.36 (0.19 – 0.68)	80 (0.4%)
	v2_6-9_AZ	122 (0.1%)	*	0.24 (0.03 – 1.69)	14 (0.1%)
	v2_10+_AZ	32,703 (27.5%)	362 (42.3%)	0.32 (0.27 – 0.39)	3,668 (16.5%)
	v3_0:1_AZ	4,596 (3.9%)	46 (5.4%)	0.21 (0.15 – 0.30)	1,584 (7.1%)
	v3_2+_AZ	1,717 (1.4%)	44 (5.1%)	0.39 (0.27 – 0.56)	1,460 (6.6%)
	v1_4+_Mo	352 (0.3%)	0 (0%)	0.00 (0.00 – Inf)	105 (0.5%)
	v2_6-9_Mo	126 (0.1%)	*	0.43 (0.06 – 3.08)	44 (0.2%)
	v2_10+_Mo	1,649 (1.4%)	*	0.04 (0.01 – 0.29)	1,382 (6.2%)
	v1_0:3_PB	839 (0.7%)	*	0.53 (0.17 – 1.65)	84 (0.4%)
	v1_4+_PB	7,655 (6.4%)	14 (1.6%)	0.33 (0.19 – 0.56)	1,172 (5.3%)
	v2_0:1_PB	236 (0.2%)	0 (0.0%)	0.00 (0.00 – Inf)	72 (0.3%)
	v2_2-5_PB	119 (0.1%)	0 (0.0%)	0.00 (0.00 – Inf)	90 (0.4%)
	v2_6-9_PB	423 (0.4%)	0 (0.0%)	0.00 (0.00 – Inf)	176 (0.8%)
	v2_10+_PB	19,293 (16.2%)	76 (8.9%)	0.18 (0.14 – 0.23)	6,930 (31.2%)
	v3_0:1_PB	1,502 (1.3%)	7 (0.8%)	0.10 (0.05 – 0.21)	614 (2.8%)
	v3_2+_PB	2,645 (2.2%)	26 (3.0%)	0.26 (0.17 – 0.40)	1,663 (7.5%)
Previous positive test	No previous positive	117,724 (98.8%)	852 (99.5%)	1	20,398 (91.9%)
	1-28 days prior	274 (0.2%)	*	0.51 (0.13 – 2.06)	0 (0.0%)
	29-90 days prior	195 (0.2%)	*	0.66 (0.09 – 4.67)	84 (0.4%)
	91+ days prior	907 (0.8%)	*	0.14 (0.02 – 1.02)	1,723 (7.8%)

Total is the total number of individuals who tested positive with each type of infection (S positive or S negative) and Hospital are the numbers admitted to hospital from the community with a S positive infection. The percentages are column percentages and sum to 100 for each characteristic, and so can be used to compare the distribution of the levels of the characteristic in the total positive cases and in the hospitalised cases. HR is the hazard ratio of admission to hospital and LCL/UCL and the lower and upper 95% confidence intervals. Hospital information is not shown for S Negative infections as the numbers are too small. The numbers in some cells have been suppressed where they are below 5 (*). Some rows of the vaccine type and status variable were omitted where there were less than 100 observations and no events – v1_0:3_AZ and 3 rows for Moderna.

Figure S1: Rate of S-gene positive and S-gene negative confirmed infections from community samples in Scotland from November 15 to December 19, 2021 by vaccine status

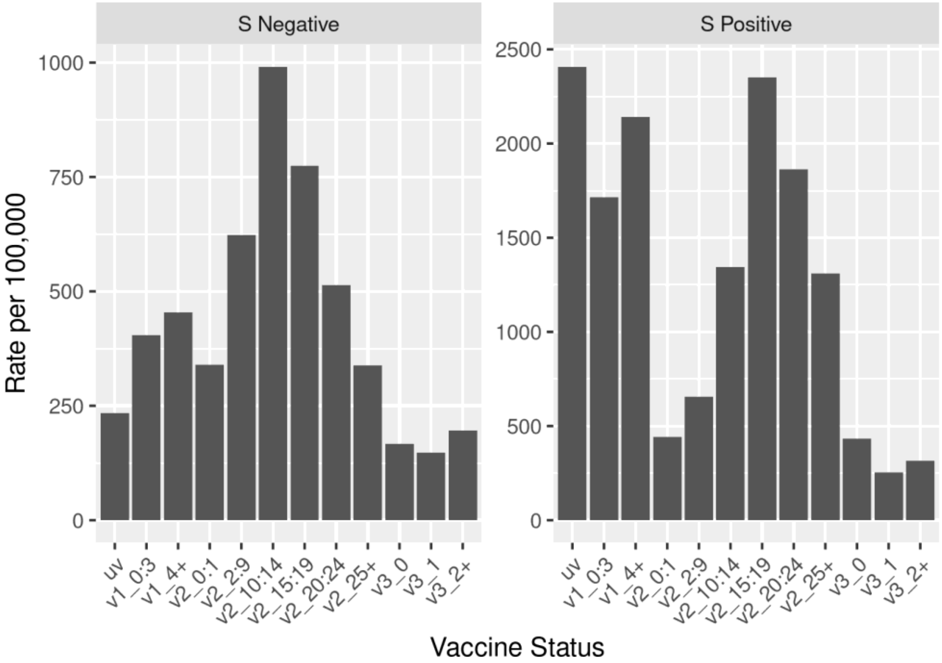

The above chart shows that the pattern of tested positive S negative infections is not the same as for S positive infections. In particular, for S positive the rate is high among the unvaccinated and low among those who have had their third/booster of who have recently received their second dose. S negative infections show high rates among those who received the second dose of the vaccine 10 to 20 weeks ago. This, in part, reflects the age distribution of those who are most commonly affected with S negative infections – those aged 20-39.

Figure S2: Rate of S-gene positive and S-gene negative confirmed infections from community samples in Scotland from November 15 to December 19, 2021 by age group

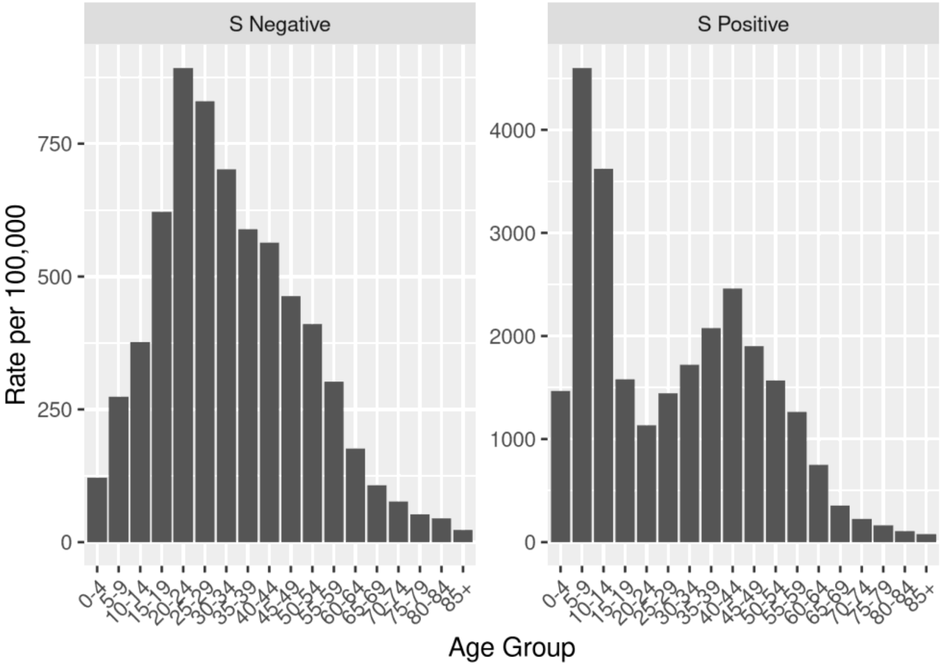

This chart shows that the age distributions of S positive and S negative infections in Scotland is quite different. The rate of S positive infections is much higher in children whereas the highest rates of S negative infections are in young adults

Figure S3: Hospital admissions within 14 days of a positive test among individuals who tested positive in the community in Scotland from November 1, 2021 by S-gene status.

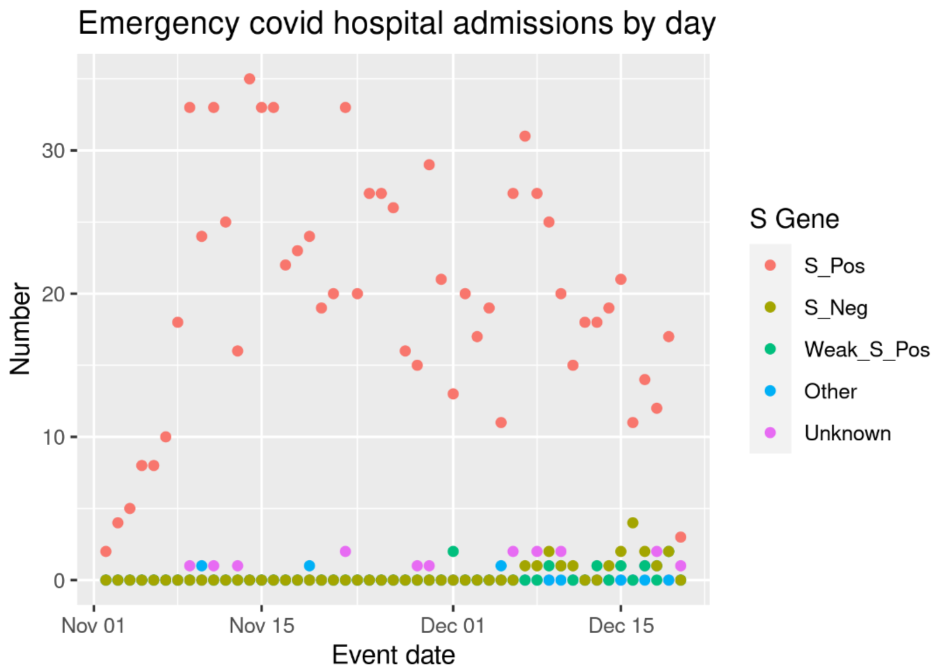

This graph counts only people who were not in hospital at the time of test. The increase in admissions at the beginning of the time period for individuals with S positive infections reflects the selection criterion of testing positive from November 1, 2021[t] and the time to hospital admission from testing positive. Individuals can be admitted any day following the positive test but most are admitted within 5-10 days following testing positive.

Figure S4: Crude rates of admission to hospital within 14 days of a positive community test by age group

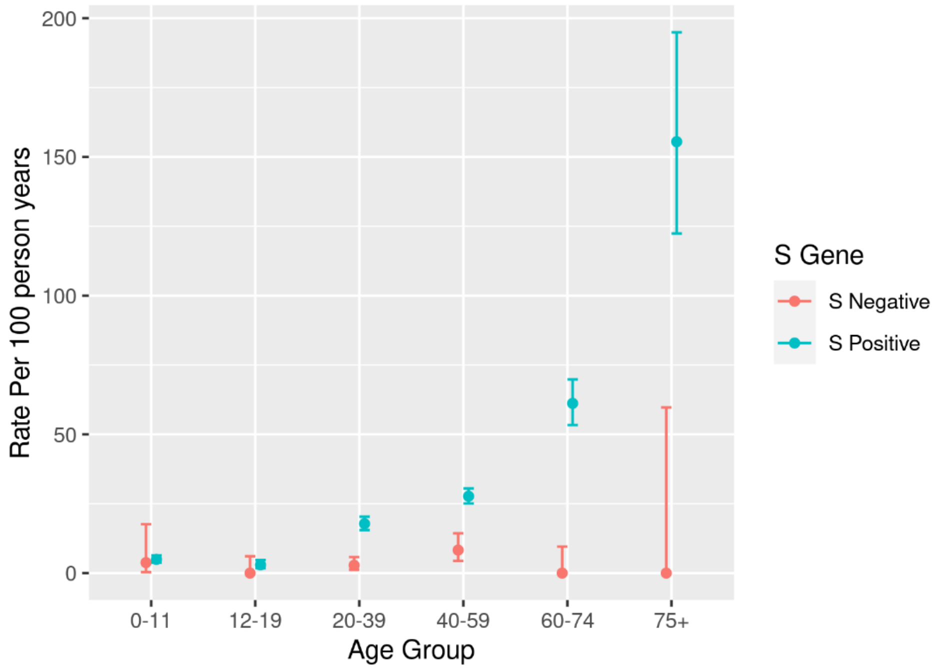

Supplement: Supplementary appendix [file mmc1.pdf]
